# Shared Experiences with Caregiving for Loved Ones Living with Dementia in an Adult Day Health and Resource Center: A Thematic Analysis

**DOI:** 10.3390/geriatrics11050129

**Published:** 2026-09-10

**Authors:** Michelle D. Hand, Megumi Inoue, Li-Mei Chen, Naoru Koizumi, Emma Booker, Sarah Nosrat, Samreen Mehak, Eyesha Shaikh

**Affiliations:** 1 College of Public Health, George Mason University, Fairfax, VA 22030, USA; minoue2@gmu.edu (M.I.); lchen38@gmu.edu (L.-M.C.); 2 Schar School of Policy and Government, George Mason University, Fairfax, VA 22030, USA; nkoizumi@gmu.edu; 3 Frederick County Department of Social Services, Winchester, VA 22601, USA; 4 Alexandria Department of Community & Health Services, Alexandria, VA 22311, USA; 5 Department of Social Work, George Mason University, Fairfax, VA 22030, USA; 6 Loudoun County Public Schools, Haymarket, VA 20148, USA

**Keywords:** caregiving, dementia, qualitative research, thematic analysis

## Abstract

**Background:** Slowing the progression of dementia and maintaining mental and social health and well-being are important aspects of health and healthcare for people living with dementia (PLwD). Relationships with family caregivers and unique caregiver insights are also important aspects of the health and well-being of PLwD. **Methods:** A group-based gaming system was developed to promote health and well-being for PLwD through movement, cognitive exercises, and social interaction. Included were family caregivers of clients living with mild- or moderate-stage impairment in an adult day center selected for the gaming intervention. Prior to implementing the intervention, three separate focus groups were conducted with 21 caregivers of PLwD, divided into each of the three focus groups based on their availability, to explore what it is like to care for someone living with dementia, their loved ones’ mood, and their questions or concerns regarding the gaming intervention. Responses were thematically analyzed. **Results:** In total, 21 caregivers participated in the focus groups. Six themes were identified: mutuality and strategies for coping with varying emotions; new challenges and successes with connecting and relating; multidimensional challenges with adjusting to decline, help-seeking, and providing care; community, social support, and resilience; technology and community resources as tools to ease caregiving burdens, and interest in digital gaming for PLwD. **Conclusions:** Our results suggest that group-based digital gaming interventions may be useful to both caregivers and PLwD, by offering meaningful opportunities for social engagement.

## 1. Introduction

Working to maintain manageable behavioral and psychological symptoms and efforts toward delaying the progression of dementia are especially important aspects of the health and healthcare for people living with dementia (PLwD) and their caregivers, who are often unpaid family members or friends [1]. In the US, roughly 83% of the provision of care to older adults is taken on by family members, friends or other caregivers who are not paid for the care they provide [1]. Approximately 48% of the caregivers who assist older adults care for people living with Alzheimer’s Disease or another form of dementia and it is estimated that 11 million people in the US are unpaid caregivers for PLwD [1].

Exploring the needs of family caregivers and advancing novel interventions to address the challenges related to caring for PLwD are critical for public health [2,3,4]. Social engagement is particularly effective in delaying dementia symptoms among people with mild cognitive impairment [5], including through game play [6], which can promote a sense of belonging and identity [4] along with improved mental health functioning [6]. Social engagement is also associated with better mood and behaviors among PLwD [6].

Concurrently, physical activities can improve both physical strength and cognitive outcomes in PLwD [7]. Thus, a group gaming system was developed to foster cognitive and social engagement. Before introducing the intervention using this gaming system, the current study explored caregiving challenges as well as the appropriateness of this gaming intervention on cognition, mood, and behavior in PLwD, based on the lived experiences and knowledge of family caregivers of PLwD.

## 2. Background

As the number of individuals affected by dementia continues to grow, the prevention and treatment of dementia will remain key areas of concern for the health and well-being of people as they age, as well as for their family members [1]. According to a report published in 2024, caregivers of PLwD provided approximately 19.2 billion hours of unpaid care, which has been valued at roughly $413.5 billion [1]. The prevalence of dementia is expected to rise to almost 14 million individuals by 2060, further impacting family members, many of whom will become unpaid caregivers [1].

Dementia generally involves a variety of symptoms that impact three key areas in the lives of PLwD: cognition, behavior, and social interactions [1]. Early-stage dementia often involves difficulty completing daily tasks, misplacing important items, along with confusion surrounding time and location owing to dysfunction in executive systems and cognitive abilities for PLwD [1,8]. Problems with communication often affect social well-being [8]. Questions and/or stories are frequently repeated, agitation and apathy are often expressed toward previous interests, and memories of past interactions or even of loved ones themselves may be lost as a result of memory loss, which can be difficult for both PLwD and their caregivers [8].

The role of caregiving for PLwD has been linked with poor mental, physical, cognitive, social and emotional health [1,2] along with positive aspects of providing care, such as mutuality, satisfaction, sense of purpose, and personal growth [3]. The experiences of family caregivers have significant implications for public policy yet remain poorly understood [9]. As Kline et al. (2025) have highlighted, “engagement of people with everyday experience of caregiving can provide important insight to inform public health programming and interventions,” [10] (p. 2). As the number of PLwD is expected to substantially increase in the US, there is a critical need to address dementia and its symptoms owing to widespread impacts on PLwD and caregivers [1,9].

People may partake in different kinds of gaming activities for entertainment and social engagement across the lifespan [11]. The digital gaming sphere has expanded extensively within the past decade. Several interventions have been implemented to evaluate the effects of gaming technologies on cognitive function and improvements among PLwD and for individuals living with other cognitive impairments [11]. Although the effects of digital gaming on cognitive stimulation have been assessed, such as enhanced alertness, empowerment, and mood, few interventions have sought to test the effectiveness of group-based digital gaming that incorporated and assessed social engagement, along with cognitive stimulation, which may yield combined improvements in mood and behavior among PLwD [12,13].

As technology will continue to develop, new digital gaming and training interventions will emerge to assist PLwD. Obie Technology is one such training tool that aims to encourage cognitive function, social interaction, and movement for older adults including PLwD [14]. While previous studies have combined portions of cognitive stimulation, physical movement, and social engagement, there is limited knowledge on how a program like Obie that intervenes to enhance all three components may affect PLwD, and whether the desired effects will be significant [15]. Enhanced cognitive stimulation, physical movement, and social engagement among PLwD may indirectly ease the daily challenges caregivers of PLwD face, such as enhancing relational interactions, supporting emotional connection, and alleviating caregiving-related stress.

This study is part of a larger intervention project examining the impact of a group-based digital gaming intervention using Obie Technology, involving roughly 30 min of group-based digital game play twice weekly for 20 weeks, following the focus group discussion with caregivers that is summarized in this manuscript. For context, the Obie equipment was purchased with grant funding that was obtained for this study (noted in the acknowledgments and funding sections). No special assistance was provided, as the equipment was user friendly. This group-based digital gaming intervention involves games being projected onto a table (e.g., Mahjong, which involves matching identical tiles by touching them, and games that involve matching countries to their flags, popping bubbles, etc.) while PLwD interact with them by “touching” the projected images on the table with their hands. Findings regarding the experiences of the caregivers following their loved ones’ participation in the gaming intervention are reported in a separate manuscript that is currently under review, which focuses on perceived impacts of the intervention after its completion. In contrast to the mixed methods manuscript, which reports the quantitative and post-intervention focus group data [15], the present manuscript reports the caregivers’ experiences prior to the implementation of the gaming intervention, focused on their caregiving experiences and challenges, as well as their interests in and questions about the gaming intervention.

The present analysis focuses on understanding caregivers’ experiences of supporting their loved ones living with early-to-moderate-stage dementia and their interests, questions, and concerns regarding the digital gaming intervention prior to its implementation. The specific aims of the study were to explore (a) their lived experiences and key challenges involved in caring for a loved one living with early-to-moderate-stage dementia, and (b) the potential appropriateness of a group-based gaming intervention to enhance cognition, mood, and behavior among their loved ones. Findings on the impact of the intervention are presented in a separate publication.

## 3. Methods

### 3.1. Research Questions

The research questions for this first (qualitative) phase of this mixed methods study were two-fold:

1. What is it like to care for a loved one who is living with dementia?
2. What (if any) interests, questions and/or concerns do caregivers have regarding the use of a digital group-based gaming intervention for improving the cognitive, mental and behavioral health of PLwD?

These questions focused specifically on the caregivers’ perspectives prior to the implementation of the gaming intervention. Questions regarding caregivers’ perceptions of intervention outcomes were examined separately after completing the intervention and are reported in a separate manuscript [15].

### 3.2. Participants and Procedure

The present study is a subset of a larger study [15] for which subject recruitment details are previously described. Participants were recruited from an adult day health center in a large East Coast county that specializes in caring for PLwD. Included in the study were caregivers of individuals living with early-to-moderate-stage dementia, defined as Montreal Cognitive Assessment (MoCA) scores of greater than or equal to 20 for early-stage dementia or MoCA scores 10–19 for moderate-stage dementia who were able to participate in the group-based digital gaming intervention activity twice weekly for 20 weeks. Family caregivers of PLwD were eligible if they provided care to a care recipient who was enrolled in the larger digital gaming intervention study. Caregivers whose loved ones did not meet intervention eligibility criteria were not eligible for the qualitative portion of this larger study.

Participants were recruited for the study from October to November, 2023, by the local adult day health center, which has three groups—early, moderate, and late groups—using purposive sampling based on predefined eligibility criteria. Specifically, the adult day center conducts the Montreal Cognitive Assessment (MoCA) to assess the level of dementia at the time of enrollment and annually after that. The goal of the parent intervention was to recruit 20 people from an early-stage group and 20 people from a moderate-stage group, and a caregiver representing each person living with dementia who was recruited to participate in the group-based digital gaming intervention.

Adult day health center staff introduced the study to eligible clients and their family caregivers and asked whether caregivers were willing to be contacted by the research team for additional information. Caregivers expressing interest were contacted by the research team by telephone to discuss the study and review the informed consent process. The consent form was then emailed to potential participants by a member of the research team to obtain their consent for their care recipient to participate in the intervention. Thus, written consent was obtained from the caregivers on behalf of their loved ones, and assent was obtained from their care recipients prior to participation.

In total, 21 caregivers representing 21 of the 24 people living with dementia enrolled in the larger intervention study participated in the focus groups. Data regarding the total numbers of individuals screened, eligible, invited, or declining participation were not maintained for this qualitative component of the larger study that is shared within this manuscript because caregiver recruitment occurred as part of the larger intervention study. Three separate semi-structured focus groups were conducted to explore what it is like to care for someone living with mild- or moderate-stage dementia with a focus on lived caregiver experiences and key challenges, as well as on their loved one’s mood, and caregivers’ questions and concerns regarding the gaming intervention and its appropriateness for enhancing cognition, mood, and behavior for people living with early-to-moderate-stage dementia. No one caregiver participated in more than one focus group. Focus groups were used because caregiving experiences are socially shared and discussions among caregivers can promote rich collective reflections on key challenges faced by caregivers and on their experiences of caregiving.

The focus groups were conducted via Zoom by the first two authors of this manuscript, who have previous experience conducting focus groups and were the project PIs, while a trained graduate student research team member also took field notes, along with the PIs. None of the researchers had previous relationships with the participants. The interview guide was developed collaboratively by the research team based on the study objectives and existing literature. The following questions were asked: (a) “Please tell us what it is like to care for your loved one with dementia, (b) What are the challenges you face?”, (c) “Can you give us an example of a situation that has been challenging to you?”, (d) “How would you describe your loved one’s mood?”, and (e) “What questions may you have for us?” This study elucidated key challenges faced by caregivers and perceptions, questions and concerns regarding the group-based gaming intervention.

Probing questions were included in addition to the semi-structured focus group questions to gather further details during the focus groups. This included, for example, asking whether participants had anything to add following the first and second questions, regarding what it is like to care for a loved one living with dementia and challenges experienced by the caregivers. The participants were also asked whether they had additional questions, with the question “What questions may you have for us?” Still, it is noteworthy that answers to questions the participants had during the focus groups may have influenced subsequent questions. The focus groups lasted approximately 60 to 90 min. At the conclusion of the focus groups, several participants independently expressed interest in connecting with one another. In consideration of this, contact information was exchanged only after obtaining the participants’ permission to share their information with other interested caregivers.

All three focus groups were recorded and transcribed via Zoom, as well as manually by trained graduate student research team members, to check for accuracy and update the transcripts as needed. Zoom transcripts served as the initial transcripts and were reviewed against the recordings and corrected manually. Field notes were used to document contextual observations and to support interpretations made during the analysis. A PI then led the thematic analysis of the focus group data. Further details on the sample are provided in Section 4.

### 3.3. Ethical Issues

Institutional Review Board (IRB) approval for this study was provided by the authors’ university (IRBNet Approval# 2017929-1; approved 13 October 2023).

### 3.4. Thematic Analysis

This study employed a qualitative descriptive design involving focus groups that were analyzed following Braun and Clarke’s steps for reflexive thematic analysis. The focus group transcripts were thematically analyzed by three trained graduate research assistants and the project PIs. Thematic analysis was used to identify answers to specific questions through constructing analytical themes that are explored across a full dataset [16]. As Braun and Clark (2006) suggested, a rigorous development of themes can yield an insightful analysis [16]. Thus, themes were identified across the dataset by reading and re-reading participants’ words, to ensure familiarization with the data, which were stored and analyzed in Microsoft Excel (version 2024), allowing the research team to remain close to the data as well as the order of the data. Initial codes were then identified using a combination of in vivo, descriptive, and value coding. Following the steps for thematic analysis described by Braun and Clark (2006), these initial codes were revised upon further review of the transcripts, then iteratively reviewed to yield additional themes, which were then further reviewed and revised, resulting in distinct categories [16]. These distinct categories were combined and refined through two more coding cycles, resulting in higher-level themes that capture the direct words of the caregivers.

### 3.5. Trustworthiness

Three members of the research team contributed to the coding and theme development, under the close supervision of the first author of this manuscript, using an inductive approach to coding. Priority was given to generating rich, meaningful themes, being mindful that there is no one correct interpretation of the data, following a reflexive thematic analytic approach. Thus, different opinions about the data were welcomed among the coders and were used to generate the nuanced themes described in Section 4. Consistent with reflexive thematic analysis, differing interpretations were understood by the research team as opportunities to strengthen the analysis. Credibility was increased through gathering information from a variety of caregivers. Additionally, transferability, dependability, and confirmability were increased by providing a detailed audit trail of the analysis process in a shared Microsoft Excel document.

## 4. Results

In total, 21 caregivers of PLwD selected for the group-based digital gaming intervention as part of the larger research project participated in these three focus groups. Three separate focus groups were conducted to accommodate the availability of the participants. These consisted of nine participants in the first focus group, seven participants in the second focus group, and five participants in the third focus group. Of the caregivers who participated in the focus groups, 17 were female (81%) and the remaining 4 caregivers were male (19%). Most caregivers were spouses of PLwD (N = 15; 71.4%). Of these spouses, the majority were wives caring for husbands (*n* = 13; 86.7%) and two were husbands caring for their wives (*n* = 2; 13.3%). Still, five caregivers were adult children of PLwD (23.8%). Of them, most were daughters (*n* = 4; 80%) either caring for their mothers (*n* = 2; 40%) or fathers (*n* = 2; 40%), and one caregiver was an adult son caring for his mother (20%). Further, one caregiver was a sibling providing care to his brother (4.8%). The ages of the PLwD that the participants cared for ranged from 64 to 97 years, with a mean age of 79.04 years. The ages of the caregivers were not collected given the scope of the study, as age was not necessary to address the research questions or analytic aims.

Written informed consent was obtained from all caregiver participants. As part of the larger intervention study, informed consent was also obtained for care recipients’ participation in the intervention, and assent was obtained from care recipients prior to participation. Although the present manuscript reports only caregiver focus group data, caregivers were recruited as part of a larger intervention study involving their family members; therefore, care-recipient consent/assent procedures pertained to participation in the gaming intervention rather than the caregiver focus groups themselves. Participants were reminded to join the focus group from a private location and were asked to maintain the confidentiality of discussions shared during the focus group.

Further details on the caregivers who participated in this study, which focus group they participated in, and their care recipients are provided in Table 1, below.

Six overarching themes were identified from the thematic analysis. These were: (a) mutuality and strategies for coping with varying emotions; (b) new challenges and successes with connecting and relating; (c) multidimensional challenges with adjusting to decline, help-seeking, and providing care (with social challenges, emotional challenges, and balance/routine as subthemes); (d) community, social support (with appreciation for one another and the study as a subtheme), and resilience; (e) use of technology and external resources to help ease caregiving burdens; and (f) interest in group-based digital gaming for PLwD. These themes are outlined with illustrative quotes in Table 2 and will be further discussed below. Because the thematic analysis for this study was conducted to explore patterns of shared meaning using an interpretive analysis rather than generating categories by frequency of participant endorsement, thematic frequencies are not reported.

### 4.1. Mutuality and Strategies for Coping with Varying Emotions

The caregivers shared that both they and their loved ones with dementia experienced varying emotions while interacting together. While some caregivers shared that their loved ones experienced a positive mood overall, many described mood changes, like unexpected shifts to anger among PLwD, that were challenging to navigate as caregivers. For example, participant 18 (P18) noted, “he wears diapers when he goes out, but he puts on his underwear first... and I said, ‘you know you, you have to switch it around,’ and he gets very angry, and shouts ‘just leave me alone.’” She added, “he starts pounding on the bed or desk.” P17 added, “she has threatened somebody with a cane and thrown water on somebody.” Still, others noted that their loved ones sometimes feel depressed instead of expressing anger outwardly.

In response to fluctuating moods and caregiving roles, some of which correlate with the decline of their loved ones, the caregivers provided insight into coping strategies they used to address burnout during their journey of caregiving. For example, P7 noted that she could relate to the overwhelm the other participants experienced and highlighted that in response, “the more I can remember it’s the disease, it’s not him, and the more I can be very present with him… just be there and try to be helpful, just try to be present... then, everything goes pretty smoothly”

The caregivers discussed their hobbies individually along with shared interests in helping their loved ones. For example, P13 shared experiences of deriving enjoyment from taking their loved one window shopping so they could relax together. The importance of relinquishing control to cope with caregiving was also underscored as being mutually beneficial. Or as P4 commented, “that river of denial is wonderful if you can float along on it... I kind of let go of the reins of control over the past couple of years,” adding, “and that’s in some ways helped and helped her be more at ease.” Thus, while diverse coping strategies were discussed, an emphasis was placed on finding activities that are mutually beneficial.

Mutually beneficial coping strategies were also required for some caregivers as a result of not being able to leave their loved ones alone. For example, P21 highlighted, “I’m not comfortable leaving him for very long, you know. I’ll go outside and walk for 45 min, or I’ll try to grab a yoga class. But I’m not comfortable leaving him at all.” Or as P16 described, “I literally can’t do much of anything, and he’s like right there hovering behind me.”

### 4.2. New Challenges and Successes with Connecting and Relating

The caregivers shared how difficult it was to encourage their loved one to show interests, become excited, or to bond after developing dementia; their loved ones’ memories were impacted and their loved ones had trouble relating to their previous lives and experiences, prior to dementia. For example, P16 reflected, “Sometimes he knows me. Other times he doesn’t know me… he tries to help, but even just washing the dishes, I end up having to clean up so much after him,” emphasizing, “but, you have to make him feel like he’s being useful… he will sit there and watch TV with me.” Somewhat similarly, P8 shared, “he’s very forgetful, and he can’t tell you what he did earlier. He repeats questions over and over.” Still, she highlighted, “But, he can have a conversation... and he can joke and laugh, and he’s still got that same twinkle... his personality is still there, but it’s kind of hard.” She added, “you’re kind of waiting for whatever the next shoe is to kind of drop and figure out how you’re going to how you’re going to deal with that. So, that’s been kind of challenging.”

Shared hobbies and interests were identified as a means of offering connection, by inviting conversation and relating to another. Still, it was noted that engagement with hobbies changed with the progression of dementia. As P18 shared, “the things he used to enjoy and do, there’s no joy.” Similarly, P16 highlighted her husband used to enjoy watching football but added, “I don’t think he understands the game anymore.” One participant noted that her husband sometimes “slips back” into using his native language as well, which she isn’t fluent in, and which hampers potential to connect through shared hobbies like hiking.

Additionally, P4 discussed an experience of taking her mother to a movie about a daughter caregiving for her mother, who was living with dementia, to connect and better understand dementia together. She did not experience the bonding she had hoped for, noting that her mother “didn’t get” the movie, and that she was thankful for this because it was “depressing.” She drew parallels to her own life, underscoring that her mother is “impossible to predict... right now, I don’t see an uphill... it’s more of a downhill slide.” Still, she concluded that she personally benefitted from watching the movie, even if her mother did not understand it.

### 4.3. Multidimensional Challenges with Adjusting to Decline, Help-Seeking, and Providing Care

Multidimensional challenges were noted, which overlapped and encompassed financial, social, emotional, and mental hardships that impact overall well-being along with the ability to care for their loved one. For example, P14 shared, “I’ve read all the books but it’s still very, very difficult, for him and for me... his business was his life, and he was very social there,” adding, “and now he isn’t really social, or he has no desire to be... he must get lonely and I feel very bad about that but I don’t know what to do about it.” As P5 put it, “it’s just really hard, because… I have to do everything,” specifying, “I took over the finances… That’s something I didn’t do… I have to help with tax prep. I have to… you know, the shopping, the cleaning, the driving… he hasn’t driven in 3 years.”

Similarly, P7 reflected, “I’m doing all the home repair and home, home maintenance and everything which is not something I’m very interested in. It has to be done. And, so, it’s hard to take on all these extra responsibilities.” As P6 put it, “you don’t realize how much your loved one did until you’re doing it all.”

New and unfamiliar challenges related to information-seeking were described as well. For example, P8 shared, “I was looking to neurologists for the answers for a lot of things, and they all often don’t do anything helpful, other than... provide a diagnosis and some medicine,” specifying, “we were looking for help in terms of, when do we take away the car keys? What kind of level care does he need? What do we do, have to do to keep him safe? What can we do to help limit the progression of this?” Subthemes of social challenges and loneliness for both PLwD and caregivers, emotional challenges and grief in response to a loved one’s decline, and challenges with maintaining balance and routines are discussed below within this theme section.

#### 4.3.1. Social Challenges and Loneliness for Both PLwD and Caregivers

A key takeaway was that many PLwD struggled with losing parts of themselves because they were unable to socialize with their previous groups or communities. As P14 pointed out, several interactions once revolved around her loved one’s career, and owing to no longer being able to continue a career as a result of dementia, her loved one became isolated. In particular, P14 shared, “his business was his life, and he was very social there. And now he isn’t really social, or he has no desire to be.” Similarly, P19 highlighted, “He’s a smart guy. He was a physician, had his own practice, had his radio show and all that,” adding that now, “he reacts with depression and anxiety.” P12 added, “he wants interaction with normal people. They don’t want to interact with people that are... [of] diminished capacity.”

Social challenges were described for both PLwD and for caregivers. For example, P21 shared, “the worst part is the loneliness... people are uncomfortable with this disease... friends and the acquaintances are uncomfortable as well... it’s a lonely disease, not only for the person going through it, but for the family as well.” Similarly, P18 described feeling lonely while providing care far from her hometown, adding, “it’s very lonely because it’s just me; I have no family here.”

#### 4.3.2. Emotional Challenges and Grief in Response to a Loved One’s Decline

Emotional challenges were described not only for the changes in behavior their loved ones experienced owing to cognitive decline, but also as a result of the nature and transition to responsibilities involved in providing care. Feeling overwhelmed was common. As P19 noted, “it’s exhausting, it’s stressful; it’s a different adventure every day.” P5 also underscored the experience of grief, sharing, “I miss the person that he used to be, because he’s not that person anymore... there’s a lot of grieving, along with being irritated all the time.” The caregivers also commonly noted experiencing guilt for becoming frustrated or mad and/or for yelling at their loved ones. For example, P18 shared, “I get very emotional... he doesn’t understand that I am giving him instructions that he cannot follow, and he cannot remember,” sharing that she has felt guilty for getting angry due to changes that have occurred since her loved one developed dementia. Further, P19 shared, “when I get mad... I shout at my husband, and I feel so, so guilty and sorry, and think ‘Oh, my gosh, why, was I like that?’” P17 added, “I get my outbursts sometimes, and then I feel bad, and think, ‘it’s not his fault,’” adding, “I cannot take it.”

#### 4.3.3. Challenges with Maintaining Balance and Routines

Caregivers found it difficult to efficiently manage time, finances, domestic chores, and other factors for themselves and for their loved ones who were living with dementia. For example, P10 shared, “I have the extra joy of balancing work with care, which is challenging… but as long as, you know, it’s just the routine, day to day, he’s happy… we have a very structured, small and routine and environment.” Frustration was commonly cited by the caregivers, along with difficulty with finding time for themselves and their personal identities outside of caregiving due to how demanding caregiving for PLwD can be.

Still, structure was noted as being especially beneficial. As P10 highlighted, “as long as it’s just the routine, day to day, he’s happy.” Conversely, navigating changes in routine was noted as a challenge. For example, P17 shared, “any type of change to routine, whether it’s because of traveling or daylight savings time can lead to some anxiety, and it manifests itself at her being a little bit irritable and asking the same questions over and over.”

### 4.4. Community, Social Support, and Resilience

The caregivers shared satisfaction with the adult day center as a source of support and with the support provided during the focus groups and through external supports, which some caregivers attributed to their resilience. Most caregivers expressed gratitude for the adult day facility, citing it as a key source of support. While unanticipated, the caregivers provided support to one another by sharing coping strategies and caregiving tips during the focus groups. As a result of sharing information and experiences of burnout along with strategies for coping during the focus groups, the caregivers expressed that they felt they had the support of one another during the focus groups and also asked to exchange information to contact each other for continued support outside of the focus groups. For example, P20 shared, “this is so appreciated. I appreciate it… I can relate,” and asked, “is it possible if people agree… to get each other’s email, if we wanted to talk to each other, go out to lunch or something since we’ve made this connection?” She added, “they also have the support group for the people at [the adult day center], and I’ve signed my husband up… the more we can kind of do without getting overwhelmed is a good thing.” Several caregivers agreed to connect.

P9 highlighted the benefits of external support as well, noting, “I do the support groups,” adding that support groups offer “a lot of information resources.” P8 also discussed the importance of support from family, particularly in long-distance caregiving contexts, explaining, “I’m 3000 miles away from him, trying to manage… financial stuff, long term care, you know, all the things… My sister is local there, which is great. So, she drops in on him a lot.”

The caregivers routinely demonstrated resilience despite expecting a downward trajectory, with some noting that they are in this together. For example, P9 concluded, “it’s been a big change, just like for everybody else, and I know, like several people have said, there’s more coming,” adding, “I’m not quite sure how we’ll handle it, but we will give it our best shot. And that’s all.” While facing caregiving challenges, P14 added, “you laugh rather than cry.”

#### Appreciation for One Another, the Adult Day Facility, and the Study

The caregivers in this study expressed gratitude for one another and for the activities offered at the adult day facility, to keep their loved ones living with dementia busy with a regular routine. Gratitude and appreciation were particularly expressed during the focus groups when validation and/or resources were provided by other caregivers and while discussing burn out related to caregiving. P20 shared, “There are places and people to talk to… and this is so appreciated… is it possible if people agree, to get each other’s email, to talk to each other, go out to lunch or something since we’ve made this connection? They also have the support group for the people at [the adult day center], and I’ve signed my husband up.”

For example, some caregivers felt validated when hearing about the emotions other caregivers were experiencing as well. And after one participant noted feeling angry with their loved one’s changes in behavior, P21 shared, “Thank you for saying that, because when [my husband] got diagnosed, I told our children, ‘you need to give me some time to understand what I’m doing, because right now, I’m in the pissed off resentful phase, thank you very much.’” P13 added, “it’s interesting to hear what different things people have trouble with.”

### 4.5. Use of Technology and Community Resources as Tools to Help Ease Caregiving Burdens

Unanticipated and unprompted by the researchers, the caregivers shared external resources with one another, with a chief focus on how technology and technological devices can be used to support their loved ones. The caregivers actively sought and exchanged information they had learned about through caregiving about technological solutions that enhanced safety and promoted the independence of their loved ones, while reducing caregiving burden. Thus, the caregivers described technology as a means of extending their ability to provide care to their loved ones while preserving the autonomy of their loved ones. In particular, Uber, Lively Mobile Plus, Go Go Grandparent, and wearable devices with GPS capabilities were recommended as useful tools for tracking and supporting their loved ones. For example, P8 shared, “I know, like so many like technical gadgety things, like, that have been, like, super helpful for me,” highlighting in particular the use of technology for promoting safety from fall risks and getting lost. Similarly, P3 noted, “I’ve learned a lot of new techniques from some of the meal delivery services, for example, Blue Apron.”

Wearable devices were found to be especially useful. For example, P9 noted that their loved one “has a fall risk, so he wears a monitor around his neck. So, you know, I’m hoping that the, the wall activity won’t be.” Moreover, P8 offered, “we’ve been using Lively Mobile Plus, which is one of the things you wear... it will give me exactly where he is,” adding, “it’s just always on so it’s just a matter of getting them to remember to wear it. I like that product a lot.”

During one focus group, P20 also asked the other caregivers if they were aware of a virtual caregiver group offered via Zoom by a community agency that is overseen by a local university, and added, “they have started a caregiver group that’s meeting once a month and if interested, I can send that information.” P8 added, “I have an echo show device, so I can pop in and hang out with him at any point in time and see what he’s doing, and chat with him,” noting that this allows her to “help kind of get him ready in the morning.”

### 4.6. Interest in Group-Based Digital Gaming for PLwD

Some caregivers shared that their loved ones enjoyed playing games. For example, P7 shared, “He’ll go for walks and like to play games... we have one caregiver [that] just love[s] to play a couple of hours games at night, so that’s really good.” Moreover, the importance of engaging in play in general was underscored. For example, P15 shared that play helps promote social engagement while keeping her loved one “occupied,” and “feeling that he’s doing something meaningful.” P13 added that she believed her husband would enjoy playing games, noting her husband “misses having the social interaction with other men.”

Further, interest was expressed in the potential for the group-based digital gaming intervention to offer enjoyment and physical activity for their loved one. For example, P21 mentioned, “I watched a video on it,” (referencing the gaming system) and then told her husband, “you should try. It looks like it’s going to be a lot of fun.” P17 also expressed hope that her mother would enjoy the group-based digital gaming intervention. Further, P21 added, “I think he would enjoy it,” referencing the group-based gaming activity, and elaborated, “I’m trying to get him more active. So, even something fun like that... if he could make it, just go in there for the game time... that might be a possibility.”

Still, a concern for potential falls during the group-based digital gaming activities was expressed. This concern was alleviated when the researchers explained that the activities are completed in a seated position on chairs while participants are supervised by members of the research team. The other concern that was expressed was that some of the loved ones the caregivers were caring for, who would be participants in the group-based digital gaming intervention, did not enjoy playing games. While noting this concern, the caregivers expressed that they were hopeful that their loved ones would enjoy the group-based digital gaming activities.

## 5. Discussion

The purpose of this study was to explore challenges with providing care to a loved one living with dementia, and to explore interests, questions and concerns regarding the group-based digital gaming intervention that was implemented based on caregiver feedback for improving the cognitive, emotional, and behavioral health of PLwD (discussed in a separate manuscript). The caregivers underscored challenges surrounding changes in relationships with loved ones, who were primarily spouses or parents, as well as changes in daily emotions and the need to develop new strategies for coping with both their loved ones’ emotions and their emotional health challenges related to decline. Notably, caregivers expressed challenges related to emotional connections and the social challenges their loved ones encountered.

With regard to the emotional health of PLwD, the caregivers highlighted multidimensional challenges pertaining to variable emotions, including anger and aggression expressed by their loved ones toward them as caregivers, and they generously shared strategies for coping with one another. Thus, while not anticipated, the caregivers made use of the focus groups to share valuable resources with one another, some of which involved learning new technology in particular. Similarly unanticipated by the research team, the caregivers expressed gratitude for the focus groups, particularly for the opportunity to share validating experiences and resources. Further, they expressed gratitude for the day center and for one another.

With regard to interests, questions and concerns surrounding the group-based digital gaming intervention for improving cognitive, emotional and behavioral health for PLwD that was discussed, the caregivers expressed particular interest in potential for group-based digital gaming to foster enjoyment and promote physical activity. Still, a concern about the potential for falling while engaging in group-based digital gaming was shared prior to the researchers responding that the group-based digital gaming activities are played while seated in chairs and supervised by members of the research team.

Adding to this, concerns were expressed that it was possible that the games would not be enjoyable for PLwD who do not enjoy game play; yet the caregivers also remained hopeful for the potential for enjoyment, including if their loved ones did not enjoy playing games in general. The games were also described as “activities” rather than “games” later during the intervention, integrating the valuable insights provided by the caregivers in this study.

### 5.1. Implications for Healthcare Practice

Considering these findings, healthcare practitioners who work with PLwD and their caregivers (e.g., social workers, nurses, and adult day facility staff) should ensure that caregivers are aware of social groups and other supports to assist them with caregiving. This can be achieved by highlighting virtual support groups held over Zoom for caregivers with limited transportation and/or for caregivers and PLwD living in rural areas. Social workers, nurses, and other healthcare professionals who work with PLwD and their caregivers should also work toward developing trainings on coping strategies that support both the health and well-being of caregivers, as well as for their loved ones living with dementia.

The results also suggest that caregivers of PLwD could benefit from resources to assist with helping to understand and process emotions in light of multidimensional caregiving challenges to help mitigate feelings of guilt for their responses to their loved ones’ decline and stressors relating to caregiving. This could improve the overall care of PLwD and as such, their health and well-being. As such, the results support a previous research recommendation to offer peer support to each other as they navigate the progression of dementia [2]. Moreover, this study’s inquiry aligns with and directly advances social work’s Grand Challenge to harness technology for good by examining caregiver interests, questions and concerns surrounding a technological advancement that can be used to improve the lives and health of PLwD and their caregivers [17].

### 5.2. Implications for Healthcare Research

The caregivers both implied and explicitly stated their challenges, during their care for PLwD, with burnout, feelings of being overwhelmed, mixed emotions, and struggles with navigating new caregiving and help-seeking challenges as they arise. Burnout and overwhelm stemmed from the caregivers providing care to their loved ones with limited opportunities or time for themselves. This led to the introduction of new technological gadgets that allowed PLwD to be monitored, which helped to reduce caregiver stress. In particular, wearable devices were of interest to the caregivers and the caregivers expressed interest in the potential for digital gaming activities to offer enjoyment while encouraging socialization and movement. Future research would benefit from further exploring the potential use of technology to support PLwD and their caregivers in promoting quality care as well as the health and well-being of PLwD.

Further research on strategies for addressing risks for caregiver burnout should be further explored as well, especially within a social work context to guide future social work interventions with caregivers, along with approaches for promoting prosocial behavior and peer support among caregivers of PLwD. The caregivers expressed particular gratitude for learning of shared experiences and for validation and resource suggestions they received during the focus groups. The potential benefits of and best practices for mutual coping strategies for caregivers should also be explored in future translational research, as this has implications for the care of PLwD and their health and wellness.

### 5.3. Implications for Healthcare Policy

Considering the unique caregiver social support and resource needs that were identified, the development of further support services for caregivers is recommended, incorporating the direct input of caregivers who regularly provide care for PLwD. Informal family caregivers should be provided opportunities for pro-social engagement with other caregivers, such as in future studies and through existing healthcare facilities as part of the ancillary service provided to patients and their families [18].

Moreover, the caregivers highlighted the role of stigma in hampering social health, or specifically, socialization and social support, both for themselves and for PLwD, suggesting that further outreach and community education is needed to reduce stigma that is linked with dementia. The results also highlight needs for further advocacy for insurance coverage for supportive technology, to track and monitor PLwD (e.g., through GPS and the ability to verbally check on PLwD), as discussed by the caregivers during the focus groups. The potential feasibility of integrating digital gaming interventions into adult day facilities should be explored, given the caregivers’ interest in this intervention for PLwD.

### 5.4. Limitations

While the original goal of the larger intervention study was to recruit 40 caregivers of PLwD, we recruited 21 caregivers, as some opted not to allow their loved ones to be research subjects, and thus did not participate in the focus groups themselves. This underscores a significant challenge when working with PLwD and recruiting for studies, which may be useful to other researchers. Despite this challenge, this study was carried out as planned. Because this study was not designed to compare experiences across caregiver relationship types or dementia stages, and owing to the small non-representative qualitative sample, comparative subgroup analyses were not conducted, which is another limitation of the non-representative sample and this study.

## 6. Conclusions

The results of this qualitative study particularly highlight the value in offering opportunities for meaningful prosocial engagement for caregivers of PLwD. Our results suggest that group-based digital gaming interventions may be useful to both caregivers and PLwD by offering meaningful opportunities for social engagement. Mutually beneficial coping strategies were especially highlighted, which warrants further exploration in future healthcare practice and research.

## Figures and Tables

**Table 1:** Key demographic information of participants and their care recipients.

Participant Number	Focus Group Cohort	Gender	Relationship to the Care Recipient	Recipient’s Level of Impairment
P1	1	Female	Non-spouse Relative	Moderate
P2	1	Male	Spouse: Husband Caring for His Wife	Early
P3	1	Male	Spouse: Husband Caring for His Wife	Moderate
P4	1	Female	Non-spouse Relative	Moderate
P5	1	Female	Spouse: Wife Caring for Her Husband	Early
P6	1	Female	Spouse: Wife Caring for Her Husband	Early
P7	1	Female	Spouse: Wife Caring for Her Husband	Moderate
P8	1	Female	Non-spouse Relative	Early
P9	1	Female	Spouse: Wife Caring for Her Husband	Early
P10	2	Female	Spouse: Wife Caring for Her Husband	Moderate
P11	2	Male	Non-spouse Relative	Moderate
P12	2	Male	Non-spouse Relative	Early
P13	2	Female	Spouse: Wife Caring for Her Husband	Early
P14	2	Female	Spouse: Wife Caring for Her Husband	Moderate
P15	2	Female	Spouse: Wife Caring for Her Husband	Moderate
P16	2	Female	Spouse: Wife Caring for Her Husband	Moderate
P17	3	Female	Non-spouse Relative	Moderate
P18	3	Female	Spouse: Wife Caring for Her Husband	Moderate
P19	3	Female	Spouse: Wife Caring for Her Husband	Moderate
P20	3	Female	Spouse: Wife Caring for Her Husband	Early
P21	3	Female	Spouse: Wife Caring for Her Husband	Moderate

**Table 2:** Summary of themes and subthemes.

Theme	Subtheme	Illustrative Quote
1. Mutuality and Strategies for Coping with Varying Emotions	*-*	“I can relate… you don’t know what you’re dealing with from minute to minute… the more I can remember it’s the disease, it’s not him, and the more I can be very present with him... then, everything goes pretty smoothly” (P7).
2. New Challenges and Successes with Connecting and Relating	*-*	“Sometimes he knows me. Other times he doesn’t know me… he tries to help, but even just washing the dishes, I end up having to clean up so much after him. But, you know, you have to make him feel like he’s being useful… he will sit there and watch TV with me” (P16).
3. Multidimensional Challenges with Adjusting to Decline, Help-seeking, and Providing Care	Social Challenges and Loneliness for Both PLwD and Caregivers.	“I’ve read all the books but it’s still very, very difficult, for him and for me... his business was his life, and he was very social there. And now he isn’t really social, or he has no desire to be... he must get lonely and I feel very bad about that but I don’t know what to do about it” (P14).
	Emotional Challenges and Grief in Response to a Loved One’s Decline.	“I miss the person that he used to be, because he’s not that person anymore. And that’s so, there’s a lot of kind of grieving along with being irritated all the time” (P5).
	Challenges with Maintaining Balance and Routines	“I have the extra joy of balancing work with care, which is challenging… but as long as, you know, it’s just the routine, day to day, he’s happy… we have a very structured, small and routine and environment” (P10).
4. Community, Social Support, and Resilience	Appreciation For One Another, The Adult Day Facility, and the Study	“There are places and people to talk to… and this is so appreciated… is it possible if people agree, to get each other’s email, to talk to each other, go out to lunch or something since we’ve made this connection? They also have the support group for the people at [the adult day center], and I’ve signed my husband up” (P20).
5. Use of Technology and Community Resources as Tools to Help Ease Caregiving Burdens	*-*	“We’ve been using Lively Mobile Plus, which is one of the things you wear... it will give me exactly where he is… it’s just always on so it’s just a matter of getting them to remember to wear it. I like that product a lot” (P8).
6. Interest in Group-based Digital Gaming for PLwD	*-*	“I think he would enjoy it… I’m trying to get him more active. So, something fun like that... if he could make it, go in for the game time... that might be a possibility” (P21).

## Data Availability

The qualitative data collected are not publicly available because participants did not provide consent for public data sharing and the narratives contain potentially identifiable personal information despite de-identification, particularly in consideration of the sample size of only 21 participants linked with a single adult day facility. This study was not preregistered owing to it being primary research. Requests for additional methodological information may be directed to the corresponding author upon reasonable request (mhand2@gmu.edu), subject to institutional ethics requirements and participant protections.
